# Complete genome sequence data of an Antarctic bacterium *Arthrobacter* sp. EM1 from the freshwater lake of the King George Island

**DOI:** 10.1016/j.dib.2023.109841

**Published:** 2023-11-23

**Authors:** Wan Shuhaida Wan Mahadi, Clemente Michael Vui Ling Wong, Kenneth Francis Rodrigues, Chui Peng Teoh, Herman Umbau Lindang, Cahyo Budiman

**Affiliations:** a Biotechnology Research Institute, University Malaysia Sabah, Jalan UMS, Kota Kinabalu, Sabah 88400, Malaysia; b Sarawak Tropical Peat Research Institute, Lot 6035, Kuching–Kota Samarahan Expressway, Kota Samarahan, Sarawak 94300, Malaysia

**Keywords:** Antarctica, Arthrobacter, Whole genome sequence, Cold-adapted bacteria

## Abstract

*Arthrobacter* sp. EM1 is a cold-adapted bacterium isolated from the Antarctic region, which was known to exhibit mannan-degrading activity. Accordingly, this strain not only promises a cell factory for mannan-degrading enzymes, widely used in industry but also serves as a model organism to decipher its cold adaptation mechanism. Accordingly, whole genome sequencing of the EM1 strain was performed via Single Molecule Real Time sequencing under the PacBio platform, followed by genome HGAP de novo assembly and genome annotation through Rapid Annotation System Technology (RAST) server. The chromosome of this strain is 3,885,750 bp in size with a GC content of 65.8. The annotation predicted a total of 3607 protein-coding genes and 65 RNA genes, which were classified under 398 subsystems. The subsystem with the highest number of genes is carbohydrate metabolism (397 genes), which includes two genes encoding mannan-degrading enzymes (endoglucanase and α-mannosidase). This confirmed that the EM1 strain is able to produce cold-adapted mannan degrading enzymes. The complete genome sequence data have been submitted to the National Center for Biotechnology Information (NCBI) and have been deposited at GenBank (Bioproject ID Accession Number: PRJNA963062; Biosample ID Accession Number: SAMN34434776; GenBank: CP124836.1; https://www.ncbi.nlm.nih.gov/nuccore/CP124836).

Specification Table

**Table:** 

Subject:	Biological sciences
Specific subject area:	Genomic and Bioinformatics
Type of data:	Complete Genome Sequence in FASTA format Table Figure
How data were acquired:	Genome sequencing was performed using Single-molecule real-time (SMRT) sequencing, developed by Pacific BioSciences (PacBio); genome assembly by HGAP3; genome annotation using the Rapid Annotation using Subsystem Technology (RAST) server.
Data format:	Raw and Analyzed
Description of data collection:	The reads of *Arthrobacter* sp. EM1 produced from the PacBio sequence platform, and *de novo* assembled into two contigs using HGAP3 assembler. The genome annotation was completed using RAST.
Data source location:	Biotechnology Research Institute, Universiti Malaysia Sabah, Kota Kinabalu Malaysia.
Data accessibility:	Repository name: National Center for Biotechnology Information (NCBI) (Bioproject ID Accession Number: PRJNA963062; Biosample ID Accession Number: SAMN34434776; GenBank: CP124836.1; https://www.ncbi.nlm.nih.gov/nuccore/CP124836).

## 1 Value of the data

• The complete genome sequence data of *Arthrobacter* sp. EM1 provides essential information and insight into the cold-adaptation mechanism of this bacterium.
• The genome data of *Arthrobacter* sp. EM1 provides the availability of more cold-adapted enzymes for accelerating knowledge and applications of cold-adapted enzymes in various industries.
• As *Arthrobacter* sp. EM1 is a type strain. The genome sequence data are useful for comparative genomic studies.

## 2 Objective

A psychrophilic *Arthrobacter* sp. EM1 was previously isolated from King George Island's Estrellas Lake (S 621°2ʹ 14.9ʹʹ W 58°57ʹ 47.5ʹʹ) in the Antarctic. It phylogenetically clustered closely with other members of the *Microbacteriaceae* family, including *Cryobacterium, Agrococcus, Frigoribacterium, Clavibacter*, and *Leifsonia*, but it also formed a distinct cluster [1]. It is worth noting that Tam *et al.* [1] also discovered that the EM1 strain exhibited resistance to several antibiotics, including ampicillin, kanamycin, and tetracycline. Biochemically, this strain has been shown to have the ability to degrade mannan-containing compounds at low temperatures, indicating the presence of genes encoding cold-adapted mannanase. Although *Arthrobacter* sp. is known to be either mesophilic or psychrophilic, there are fewer reports of psychrophilic *Arthrobacter* compared to mesophilic ones. Therefore, it is intriguing to explore the molecular insights on the cold-adaptation mechanisms of psychrophilic *Arthrobacter* sp. However, there are not many reports available on the complete genome sequences of cold-adapted *Arthrobacter* strains. This limitation hinders our ability to gain a comprehensive understanding of their mechanisms for cold adaptation. The present study aims to provide the complete genome sequence of EM1, which is usable for further comparative genomic studies aimed at understanding the mechanisms by which the group adapts to extreme temperatures and exploring beneficial genes for industrial applications.

## 3 Data Description

The Antarctic region is known for its extreme conditions, such as low temperatures, salinity, elevated UV radiation, and low nutrient and water content, which provide a unique environment for the discovery of extreme microorganisms with unique adaptation mechanisms [2]. In particular, previous studies have shown that Antarctic lakes have a surprising degree of microbial diversity, making them a promising area for the exploration of unique microorganisms [3]. The *Arthrobacter* sp. EM1 is therefore believed to possess unique genomic features related to its adaptation mechanism to the Antarctic temperature and habitat. This study presents the complete genome sequence of the EM1 strain of *Arthrobacter* sp., which was obtained using the PacBio RSII sequencing platform. The genome size of *Arthrobacter* sp. EM1 is 3885,750 bp with a GC content of 65.8%. This size was determined by comparing it to the genome sizes of other *Arthrobacter* strains, which range from 3.8 to 5.3 Mbp on average [4,5]. The assembly of the genome sequence of *Arthrobacter* sp. EM1 into three contigs with reference lengths of 23 kbp, 171 kbp, and 3.9 Mbp. The 3.9 Mbp contig was considered to be the expected genome size of *Arthrobacter* sp. EM1, as it falls within the size range of other reported *Arthrobacter* sp. genomes, which range from 3 to 5 Mbp [5,4]. The detailed genome features of the EM1 strain are summarised in Table 1.

**Table 1:** Assembled sequence statistics of Arthrobacter sp. EM1.

Genome Feature	Value
Genome length (bp)	3885,750
G+C content (%)	65.8
Number of Subsystems	398
Number of protein-coding genes	3607
Total number of RNA genes	65
rRNA genes (5S, 16S, 23S)	8
tRNA genes	15
ncRNA genes	3
Pseudo genes	1070
N50	3885,750
L50	1

**Fig. 1:**
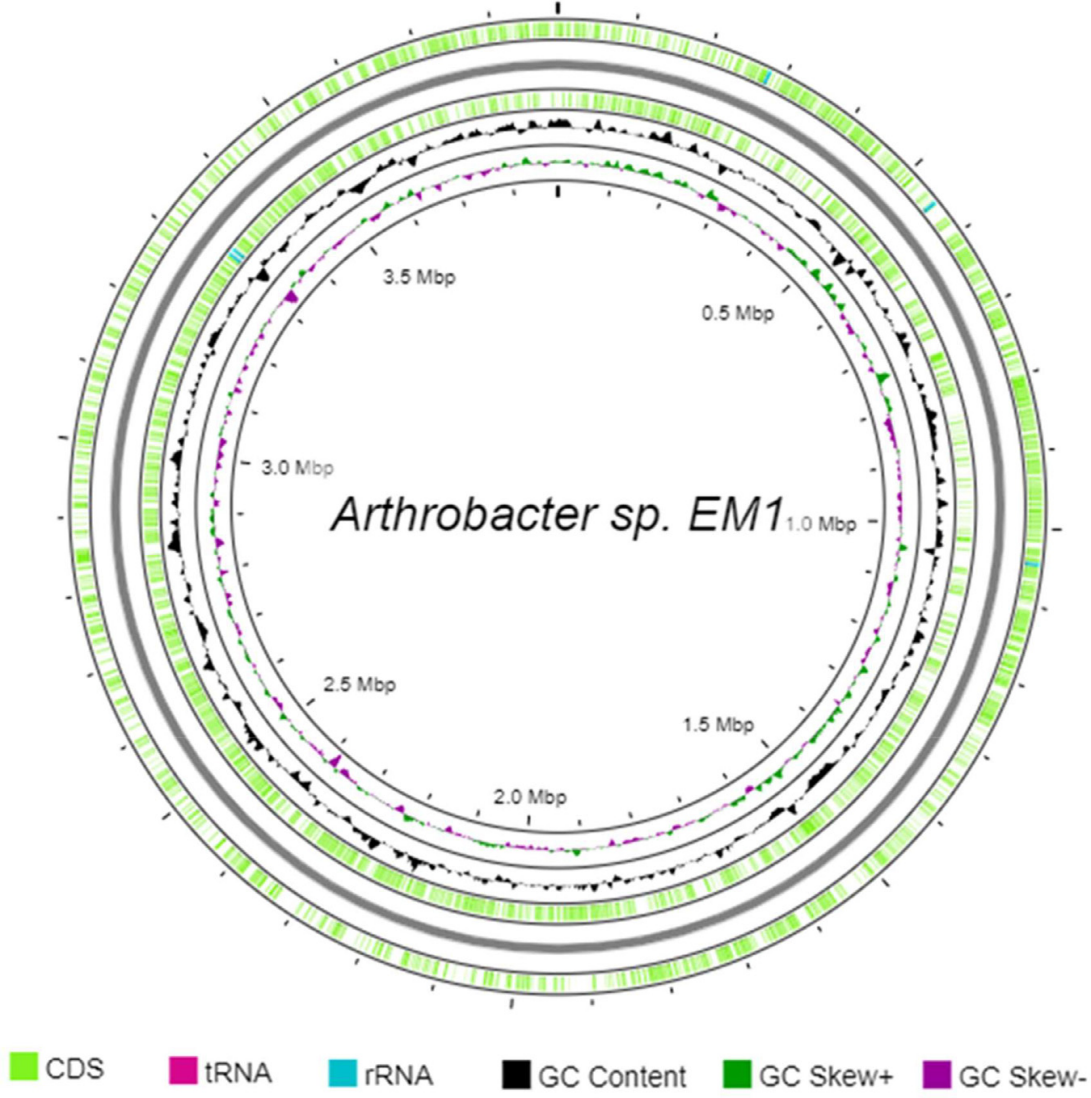
Graphical circular map of the genome of *Arthrobacter* sp. EM1.

As shown in Table 1, a total of 3607 protein-coding genes and 65 RNA genes were predicted. The genomic features of this train are summarized in Fig. 1.

**Fig. 2:**
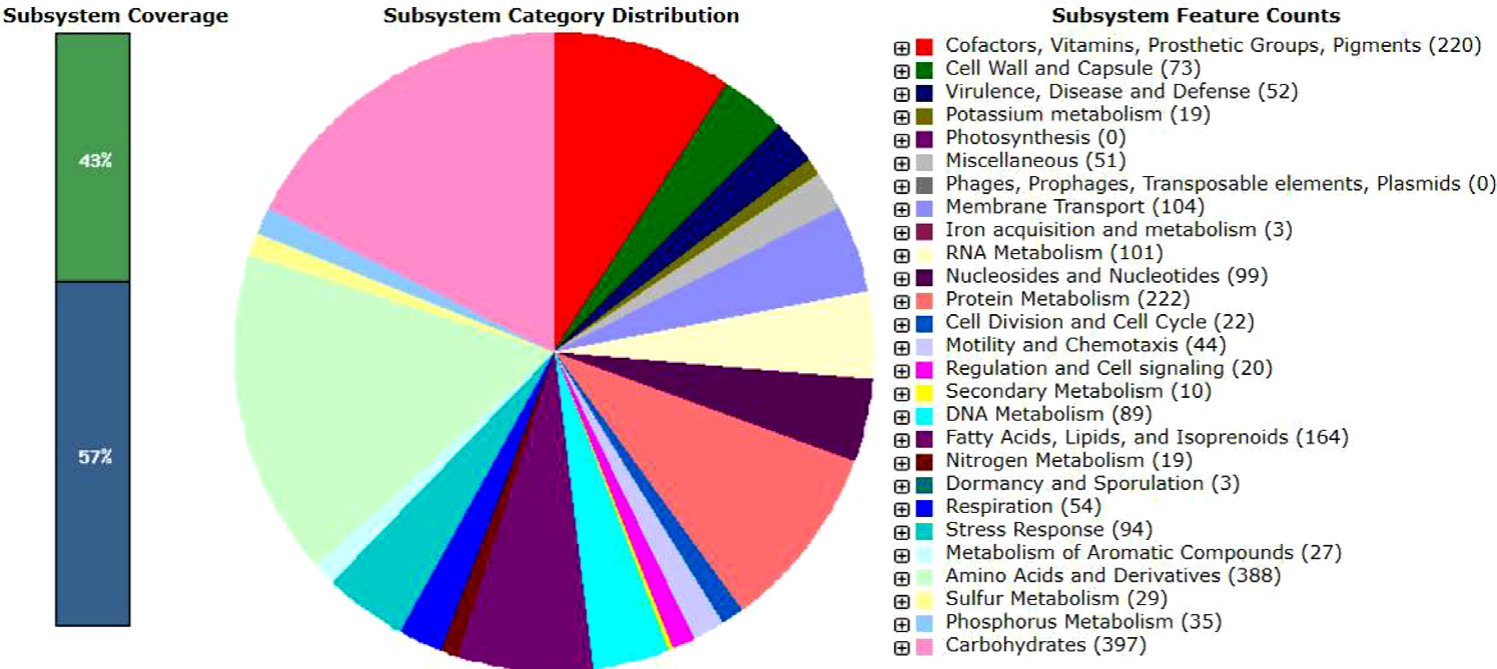
An overview of the subsystem categories of *Arthrobacter* sp. EM1. Genome annotation was conducted using the RAST server.

In addition, a total of 398 subsystems were classified with 43% of subsystems coverage (Fig. 2). Subsystem features belonged mostly to carbohydrates (397 genes); followed by amino acids and derivatives (388 genes); protein metabolism (222 genes); cofactors, vitamins, prosthetic groups, and pigments (220 genes); and fatty acids, lipids and isoprenoids (164 genes) (Fig. 2). To note, among 397 genes under carbohydrate metabolism, 26 and 12 genes were found involved in pyruvate metabolism (acetyl-CoA, acetogenesis from pyruvate) and TCA cycle, respectively. The presence of these genes indicated that the EM1 strain indeed heavily relied on aerobic metabolism for generating ATP from sugar compounds. This is also supported by the presence of 17 genes for The Entner–Doudoroff (ED) pathway, which is a major pathway of glucose catabolism under aerobic conditions [6]. This is in good agreement with a previous report indicating that members of the genus *Arthrobacter* are typically considered obligate aerobic bacteria, with only a few strains reported to be anaerobic [7]. It is worth noting that the EM1 strain was isolated from a freshwater lake, known to contain relatively high dissolved oxygen level (68.6%) [8]. The presence of the genes in glucose catabolism pathway in EM1 suggests its ability to utilize the glycolytic pathway at the low-temperature environment of the Antarctic region. Notably, these genes are also found in other Antarctic bacteria and fungi, where they are predicted to play important roles in their cold adaptation mechanisms [8,9]. To cope with oxidative stress at low temperatures, Antarctic microorganisms modulate the activity of several key enzymes in the glycolytic pathway and the TCA cycle. These enzymes include, but are not limited to, hexokinase, glucose-6-phosphate dehydrogenase, glyceraldehyde-3-phosphate dehydrogenase, isocitrate dehydrogenase, succinate dehydrogenase, and malate dehydrogenase [9].

Nevertheless, this strain is also apparently capable of exhibiting the fermentation process, as shown by the existence of 55 genes for fermentation metabolism under the carbohydrate subsystem. Further into the detail, genes under fermentation metabolism indicated that this strain is able to produce butanol (20 genes), acetolacetate (3 genes), lactate (3 genes), butyrate (24 genes), acetoin, butanediol (5 genes). These are industrially relevant compounds. The EM1 strain is promising to be further applied to produce those compounds. The presence of fermentation genes also indicated that the EM1 strain might be a facultative anaerobic bacterium which is able to switch the metabolism to anaerobic metabolism when oxygen is absent. Of note, under the subsystem of carbohydrate metabolism, 2 genes encoding mannose-metabolism-related enzymes are present, which are endoglucanase (8148 bp) and α-mannosidase (3024 bp). These enzymes confirmed that the EM1 strain is indeed a producer of cold-adapted mannan degrading enzymes. These enzymes are believed to be cold adapted enzymes, which are predicted to support the adaptation of EM1 strain at low temperature by facilitating efficient hydrolyisis of mannan-based substrate as one of carbon source at low temperature. In general, molecular basis of cold adaptation of cpyschrophilic organisms involve production of cold-evolved enzymes that are partially able to cope with the reduction in chemical reaction rates induced by low temperatures [10].

## 4 Experimental Design, Materials and Methods

### 4.1 DNA extraction

*Arthrobacter* sp. EM1, which was previously isolated by Tam *et al.* [1] and kept as a stock culture at the Biotechnology Research Institute, Universiti Malaysia Sabah, was first grown in Luria Bertani (LB) medium (Difco, US) in an orbital shaker at 15°C with 200 rpm shaking speed. Genomic DNA (gDNA) was extracted using the Qiagen Qiamp DNA Mini Kit (Qiagen, Germany) following the pre-treatment protocol for Gram-negative bacteria. Quantification and qualification of the extracted gDNA were performed using a Nanodrop DNA spectrophotometer (GE Healthcare), Qubit® fluorometer (Thermofischer), and Pippin Pulse™ Electrophoresis. The extracted gDNA was then used for library construction under a Single Molecule Real-Time Sequencing Platform (SMRT) .

### 4.2 Library preparation, sequencing and annotation

The gDNA obtained from above extraction was firstly sheared using the g-TUBE (Covaris) following the manufacturer's protocol. SMRTbell libraries were created using the ‘Procedure & Checklist—20 kb Template Preparation using BluePippin™ Size Selection’ protocol [11], which was then sequenced under PacBio® RS II system (Pacific Biosciences, CA, USA). The raw sequence data were preassembled and de novo assembled using Hierarchical Genome Assembly Process (HGAP) in Pacific Bioscience SMRT Portal (version 2.1.1.) [12]. Gene prediction was accomplished on the Rapid Annotation using the Subsystem Technology SEED viewer (RAST; http://rast.nmpdr.org/) using Subsystem Technology (RAST), with FIG's "Subsystem Approach" [13]. The complete genome sequence was then subjected to nucleotide blast using BLAST+ 2.6.0.

## Ethic Statements

No human subjects, living animals and social media platforms were involved for the data collection in this study.

## CRediT authorship contribution statement

**Wan Shuhaida Wan Mahadi:** Investigation, Formal analysis, Writing – original draft. **Clemente Michael Vui Ling Wong:** Conceptualization, Supervision, Methodology, Writing – review & editing. **Kenneth Francis Rodrigues:** Conceptualization, Supervision, Methodology, Writing – review & editing. **Chui Peng Teoh:** Data curation, Validation, Writing – review & editing. **Herman Umbau Lindang:** Data curation, Validation, Writing – review & editing. **Cahyo Budiman:** Conceptualization, Supervision, Methodology, Writing – review & editing.

## Data Availability

Arthrobacter sp. EM1 chromosome, complete genome (Original data) (NCBI GenBank)
